# Aberrant Prestimulus Oscillations in Developmental Dyslexia Support an Underlying Attention Shifting Deficit

**DOI:** 10.1093/texcom/tgaa006

**Published:** 2020-03-23

**Authors:** Lars Meyer, Gesa Schaadt

**Affiliations:** 1 Research Group “Language Cycles”, Max Planck Institute for Human Cognitive and Brain Sciences, Leipzig 04103, Germany; 2 Clinic of Cognitive Neurology, Medical Faculty, University Leipzig, Leipzig 04103, Germany; 3 Department of Neurology, Max Planck Institute for Human Cognitive and Brain Sciences, Leipzig 04103, Germany

**Keywords:** alpha band, dyslexia, mismatch negativity, neural oscillations, prestimulus activity

## Abstract

Developmental dyslexia (DD) impairs reading and writing acquisition in 5–10% of children, compromising schooling, academic success, and everyday adult life. DD associates with reduced phonological skills, evident from a reduced auditory mismatch negativity (MMN) in the electroencephalogram (EEG). It was argued that such phonological deficits are secondary to an underlying deficit in the shifting of attention to upcoming speech sounds. Here, we tested whether the aberrant MMN in individuals with DD is a function of EEG correlates of prestimulus attention shifting; based on prior findings, we focused prestimulus analyses on alpha-band oscillations. We administered an audio–visual oddball paradigm to school children with and without DD. Children with DD showed EEG markers of deficient attention switching (i.e., increased prestimulus alpha-band intertrial phase coherence [ITPC]) to precede and predict their reduced MMN—aberrantly increased ITPC predicted an aberrantly reduced MMN. In interaction, ITPC and MMN predicted reading abilities, such that poor readers showed both high ITPC and a reduced MMN, the reverse being true in good readers. Prestimulus ITPC may be an overlooked biomarker of deficient attention shifting in DD. The findings support the proposal that an attention shifting deficit underlies phonological deficits in DD, entailing new opportunities for targeted intervention.

## Introduction

Reading and spelling are critical social and cultural skills for successful schooling, academic success, and everyday adult life. The most frequent impairment in reading and writing acquisition is developmental dyslexia (DD), causing reading and/or spelling problems in 5–10% of children across literate societies (Shaywitz et al. 1990; Katusic et al. 2001; Lyon et al. 2003; Schulte-Körne and Remschmidt 2003).

Problems in reading and/or spelling are the most prominent symptom of DD, but different underlying deficits are under discussion. One view postulates an underlying phonological deficit in the processing and/or representation of speech sounds (Snowling 1998; Goswami 2015; Peterson and Pennington 2015), based on reports of deficient phoneme identification and discrimination (Mann and Liberman 1984; Snowling 1998; Moll et al. 2014). Yet, phonological deficits may also reflect a more general auditory timing deficit, because specific difficulties in the processing of quickly varying sounds predict phonological deficits (Tallal 1980; Tallal et al. 1993; Boets et al. 2008; Lallier et al. 2010; Lehongre et al. 2011). In addition, it has been argued that auditory timing deficits are really secondary to an attentional deficit (Galaburda et al. 1994; Hari and Renvall 2001; Krause 2015) Deficient attention shifting to upcoming stimuli results in an atypical perception of rapid stimulus sequences (Lallier et al. 2010), surfacing as deficient phonological decoding (Facoetti et al. 2010). Also, deficient attention shifting in DD is a cross-modal impairment, specifically affecting the switch from written text to speech sounds (Franceschini et al. 2012; Gori et al. 2016; Franceschini and Bertoni 2019).

Because of the long-term social and cultural consequences of DD, early diagnosis and intervention are critical. The electroencephalogram (EEG) has proven potential for diagnosis even prior to reading onset. The phonological deficit of individuals with DD is indexed by a decreased auditory mismatch negativity (MMN; Näätänen et al. 1978; Corbera et al. 2006; Paul et al. 2006; Lovio et al. 2010; Schulte-Körne and Bruder 2010; Schaadt et al. 2015; Männel et al. 2017; Volkmer and Schulte-Körne 2018). Yet, in spite of the proposal of an underlying attention shifting deficit, it is unclear whether MMN decreases are secondary to a failure to shift attention to upcoming stimuli. Data from healthy participants suggest MMN amplitude to decrease under distraction (Woldorff et al. 1991; Oades and Dittmann-Balcar 1995; Woldorff et al. 1998). Moreover, the neuronal sources of the MMN are not only restricted to auditory cortices in the superior temporal gyri (Escera et al. 2003; Restuccia et al. 2005), but also include brain areas known to modulate deviance detection by top-down attentional shifting, such as the inferior frontal gyrus (Marco-Pallarés et al. 2005; Garrido et al. 2008) and anterior cingulate cortex (Waberski et al. 2001). Furthermore, auditory discrimination dysfunctions associate with an impaired frontal attention-shifting mechanism (Sato et al. 2003).

We employed an audio–visual oddball paradigm to dissociate EEG correlates of deficient attention shifting from the reduced MMN in DD. To assess attention shifting independently of the MMN, we focused on prestimulus alpha-band oscillations. We decided to focus on the alpha band based on the prior reports of alpha-band synchronization during successful detection of upcoming visual and tactile targets (Hanslmayr et al. 2007; Weisz et al. 2014); auditory discrimination confidence was found to be predicted by alpha-band synchronization as well (Wöstmann et al. 2019). This fosters the hypothesis that reduced auditory MMN responses in individuals with DD may be preceded by an increase in alpha-band synchronization (i.e., reduced desynchronization) prior to stimulus occurrence. We compared school children with DD to age-matched controls. The experiment involved visually presented mouth movements forming the syllable /pa/; concurrently, we presented auditorily the congruently produced syllable /pa/ as a standard and the mismatching syllable /ga/ as a deviant stimulus; standard and deviant were swapped in a second experimental block. We hypothesized increased prestimulus alpha-band phase synchronization in the DD group compared with controls, irrespective of whether the subsequent stimulus was a standard or a deviant syllable. Prestimulus alpha-band phase synchronization should also predict MMN amplitude.

## Materials and Methods

### Participants

Data from 53 participants were included in the study. The sample consisted of 28 dyslexic participants (18 females; two left-handed; mean age = 9.69 years, standard deviation (SD) = 0.50 years) and 25 control participants (16 females; two left-handed; mean age = 9.85 years, SD = 0.56 years). Participants did not suffer from neurological or hearing deficits, had normal or corrected-to-normal eyesight, and were naïve as to the purpose of the study. Parents of participating school children were reimbursed (€7.00 per hour). The study followed the American Psychological Association standards in accordance with the declaration of Helsinki and was approved by the ethics committee of the medical faculty of the University of Leipzig.

#### Standardized Cognitive Testing

At preschool age (mean age = 5.02 years, SD = 0.06 years), children had been screened for prerequisites of literacy using the standardized Bielefeld Screening for the Early Recognition of Reading and Spelling difficulties (BISC; Jansen et al. 2002; phonological awareness, attention, phonetic recoding in short-term memory, and recall from long-term memory). According to the test, children are considered at risk of later literacy problems when scoring below the 16th percentile in at least four of the eight subtests (i.e., risk score ≥ 4).

During primary school grade 3 or 4 (mean age = 9.77 years, SD = 0.54 years), children were tested for their orthographic abilities using the German Spelling Test (DERET; Stock and Schneider 2008). Children consecutively wrote down 10 sentences from dictation, without time constraints. Spelling errors were defined as at least one spelling error within one word; performance quantification was based on the comparison to age-normed percentile ranks (PRs; Stock and Schneider 2008). Children’s phonemic awareness was assessed via dedicated subtests (e.g., phoneme deletion and phoneme exchange) of the German test for Reading and Spelling skills (BAKO; Stock et al. 2003); again, age-specific norms serve to calculate PRs. To control the verbal measures at the age of acquisition for children’s nonverbal intelligence, we used the dedicated subtests (e.g., spatial working memory and matrices) from the German version of the Kaufman-Assessment Battery for Children (K-ABC; Kaufman 2009); performance was translated into age-normed standard scores.

Two years afterward (mean age = 12.82 years, SD = 0.60 years), children were tested for their reading speed and comprehension with the Reading Speed and Comprehension Test (LGVT, grades 6–12; Schneider et al. 2007). Children silently read a cloze text as far as possible in 4 min, inserting missing words by choosing from three semantically different options. Reading speed (i.e., number of read words) and comprehension performance (i.e., number of semantically correct insertions) are translated into PRs according to the grade-specific norms.

All results of the standardized cognitive testing are provided in Table 1. DD individuals showed an early increased DD risk as well as decreased verbal performance across tests. After correction for multiple comparisons, there was a trending group difference in nonverbal intelligence. As individuals with DD should, by definition, not show reduced nonverbal intelligence (Schulte-Körne 2014; Peterson and Pennington 2015), we controlled subsequent analyses for this undesired trend.

**Table 1:** Results of standardized cognitive testing

	Control group		DD group		Difference		
	Mean	SD	Mean	SD	*t*	df	*P*
BISC	1.18	1.06	3.56	1.71	−6.17	51	<0.001*
DERET	63.64	19.52	11.24	7.57	12.60	51	<0.001*
BAKO	65.86	0.50	26.15	0.49	5.60	51	<0.001*
LGVT (speed)	46.82	24.67	27.86	18.42	2.94	51	0.005*
LGVT (comprehension)	52.07	25.77	31.38	21.07	2.98	51	0.005*
K-ABC	114.79	6.24	109.96	7.68	2.52	51	0.02

BISC, preschool risk assessment of phonological awareness; DERET, orthography assessment; BAKO, assessment of phonological awareness; LGVT, assessment of reading abilities; K-ABC, assessment of nonverbal intelligence; DD, developmental dyslexia.

^*^ *P* < 0.05, Bonferroni corrected.

### Materials

**Figure 1:**
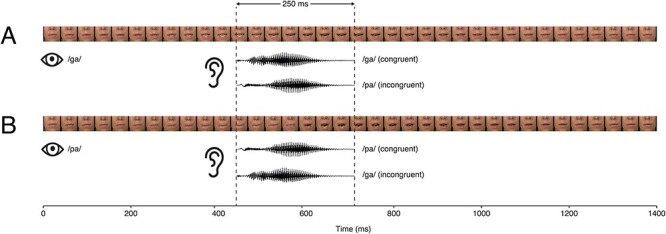
Stimulation: video frames of each syllable and waveforms of each syllable are shown for the standard and deviant conditions for the two experimental blocks.

A passive audio–visual oddball paradigm was constructed from the syllables /pa/ and /ga/, because the phonemes /p/ and /g/ distinguish between different word meanings in German (e.g., /platt/−*flat* vs. /glatt/–*slippery*) and have been shown to be discriminable both auditorily and visually (Schaadt et al. 2015, 2016, 2019). Children were presented with a video of a frequently occurring standard mouth movement while hearing either the congruently produced syllable or a mismatching syllable. For the videos, the mouth region from nose to chin of a native German actress was filmed while simultaneously recording her speech. Videos were edited in Adobe Premier Pro (Adobe Inc., San Jose, CA) to a resolution of 720 × 576 pixels at 25 frames/s. Each of the two syllables extended for 36 frames. Central mouth position on the monitor did not differ between syllables. Speech recordings were sampled at 16 bits with a frequency of 44.1 kHz. Auditory syllables started 0.45 s after video onset and lasted for 0.25 s; in total, each stimulus lasted for 1.44 s (see Fig. 1).

#### Procedure

Children and their parents were orally informed about the procedure. Children were asked to provide consent to participate and parents gave written consent on behalf of their children. The experiment took place in an electrically shielded, sound-attenuated, dimly lit EEG cabin. The child was seated in a comfortable chair. The child was instructed to carefully watch the mouth movements, which were presented on a 15-inch monitor (resolution: 1024 × 768) at a distance of ~75 cm. Auditory stimuli were presented via loudspeakers at an intensity of 64 dB sound-pressure level. Speakers were located on the left and right side directly next to the monitor placed in front of the participants at an angle of 18°. The design involved two blocks. In the one block, the mouth movement of the syllable /pa/ with the congruent auditory syllable was the standard stimulus, while the movement of the syllable /pa/ with the incongruent syllable /ga/ was the deviant stimulus; in the other block, the mouth movement of the syllable /ga/ with the congruent auditory syllable was the standard, while the movement of the syllable /ga/ with the incongruent auditory syllable /pa/ was the deviant. Block order was counterbalanced across participants. A single block consisted of 400 stimuli, with 320 standard (80%) and 80 deviant (20%) stimuli, resulting in a total of 800 stimuli. Within the sequence of standards, deviant occurrence was pseudo-randomized, such that at least two subsequent standards were presented between two deviants. The interstimulus interval (ISI) between visual mouth movements was 0.5 s, which was filled by a fixation cross to minimize eye movements. The ISI between the auditory syllables was 1.69 s. To evaluate whether children were fixating at the mouth movements, they were asked confirmation questions after the experiment (i.e., “Did you watch the mouth throughout the whole experiment?” and “Did you see differences between mouth movements?”). In addition, an observer who was unaware of children’s group status monitored each child during the experiment and rated their overall fixation at the end of each experimental block. We only included datasets in further analyses when children reported to have watched the mouth movement throughout the experiment, they mentioned to have noticed differences between mouth movements, and the independent observer rated the overall fixation of mouth movements to be >80%. Children with (mean = 92.28%; SD = 5.71%) and without DD (mean = 93.75%; SD = 4.87%) did not differ concerning their overall fixation of mouth movements (*t*(51) = 1.01; *P* = 0.32). Each block lasted 18 min with a between-block break of variable duration. Taken together, the experiment lasted ~ 40 min.

**Figure 2:**
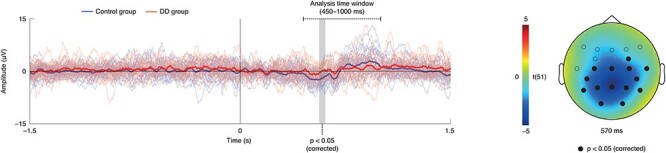
Result of analysis of ERPs: left: individual and group-average ERPs at the peak electrode (i.e., PZ; blue = control participants/group, red = DD individuals/group); the analysis time window reaches from the onset of the audio–visual mismatch until 1 s; MMN amplitude is reduced in the DD group; right: statistical difference between DD and control groups at the peak time point (i.e., 0.57 s); solid circles mark electrodes where the group difference was significant at *P* < 0.05 (corrected).

#### Data Acquisition

The EEG was recorded at 500 Hz from 23 Ag/AgCl electrodes mounted in an elastic cap according to the international 10–20 system (Easy Cap GmbH, DE; F7, F3, FZ, F4, F8, FC3, FC4, T7, C3, CZ, C4, P7, CP5, CP6, T8, P3, PZ, P4, P8, O1, O2, M1, and M2). Electrodes were referenced to CZ; an additional electrode at FP1 served as common ground. Electrooculograms were recorded bipolarly from supraorbital and infraorbital sites at the right eye, as well as from electrodes located at the outer canthi of each eye. Impedances were kept <10 kΩ.

#### Data Analysis

Data analysis was carried out using the FieldTrip toolbox for M/EEG analysis (Oostenveld et al. 2011) running in MATLAB (The MathWorks, Inc., Natick, MA). To remove slow drifts, raw EEG data were filtered with a 6th-order two-pass Butterworth infinite-impulse-response 0.5-Hz high-pass filter. Epochs of 1 s pre-onset duration plus 1.5 s postonset duration, time-locked to the onset of the stimulus, were extracted from the data. Standard trials occurring after deviant trials were discarded from further analysis. The data were then re-referenced to the mean of the mastoid channels (i.e., sensors A1/2), which were then discarded from subsequent analysis. For the detection of muscle artifacts, we employed a semi-automatic distribution-based approach that automatically identified artifacts z ≥ 5 within a pass band of 100–120 Hz (Oostenveld et al. 2011). Artifacts were manually rejected based on the visual inspection of waveform morphology. On average, 23.04% (SD = 9.31%) of data were rejected. The rejection rate differed between groups (DD: mean = 25.55%, SD = 9.57%; controls: mean = 20.81%, 6.99%; *t*(51) = −2.07, *P* = 0.04), which was taken into account by control analyses (see below). Blinks and eye movements were then corrected using independent-component analysis (Makeig et al. 1996); to-be-rejected components were identified through visual inspection of component topography and waveform; on average across participants, 2.8 components (SD = 0.9 components) were removed from the data. The number of rejected components did not differ between groups (DD: mean = 2.88 components, SD = 0.93 components; controls: mean = 2.71 components, SD = 0.90 components; *t*(51) = −0.66, *P* = 0.51).

For the analysis of the MMN event-related brain potential (ERP) to the audio–visual stimulus mismatch, the preprocessed EEG data were averaged across trials separately within the standard and deviant conditions. ERPs were corrected for baseline activity by subtracting the average potential across the time window from −0.25 to 0 s prior to the onset of the auditory information. The MMN was then calculated by subtracting the ERP to the deviant condition from the ERP to the standard condition.

For the analysis of alpha-band increased prestimulus alpha-band intertrial phase coherence (ITPC) prior to the audio–visual stimulus mismatch, we performed time–frequency analysis in 50-ms time steps across the pre-onset time window from −0.5 to 0 s at five log-spaced center frequencies across the 8–12-Hz frequency band (i.e., 8.00, 8.80, 9.60, 10.80, and 12 Hz). We employed Morlet wavelets with a fixed time–frequency resolution of seven cycles; from the complex output, we then calculated ITPC (Tallon-Baudry et al. 1996; Lachaux et al. 1999). To focus on participants’ prestimulus attention to the upcoming stimulus (i.e., irrespective of the eventual stimulus category, standard or deviant), ITPC was calculated across all trials (i.e., standard and deviant).

For statistical comparison of the MMN ERP and alpha-band ITPC between dyslexics and controls, we employed nonparametric cluster-permutation independent-samples *t*-tests. These identified significant time–electrode/time–frequency–electrode clusters (MMN ERP/ITPC, respectively) while controlling for false positives (Maris and Oostenveld 2007; *P* < 0.05, α = 0.05, 10 000 permutations, ≥3 channels minimum cluster size). For both dependent measures, we chose to assess the cluster-sum statistic. For the MMN ERP, statistical analysis was carried out from 0.45 to 1 s (i.e., the time window after the onset of the audio–visual stimulus mismatch); for alpha-band ITPC, statistical analysis was carried out from −0.5 to 0 s (i.e., the time window before the onset of the visual mouth movement).

## Results

### MMN ERP and ITPC

The comparison of the MMN ERP between the DD and control groups revealed a single significant cluster (cluster-sum *t*(51) = −576.16, cluster-level *P* = 0.04; peak level *t*(51) = −4.95, *P* = 0.039) in the time window from 0.56 to 0.61 s with a broad scalp distribution (channels FC4, CZ, C3, C4, T8, CP5, CP6, PZ, P3, P4, P7, P8, O1, and O2; Fig. 2) where MMN amplitudes were reduced for dyslexic relative to control participants. Given the late onset of the audio–visual mismatch (i.e., 0.45 s after stimulus onset), we interpret this effect as a reduced MMN ERP for dyslexic relative to healthy participants.

**Figure 3:**
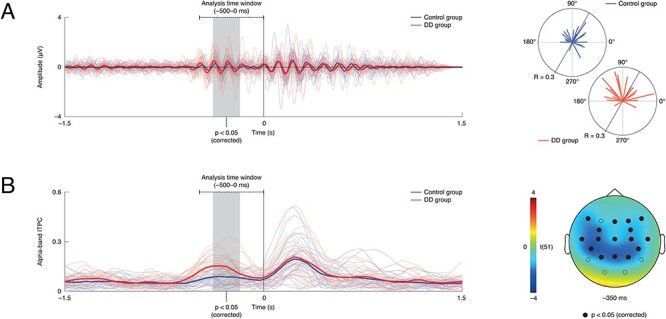
Result of analysis of prestimulus alpha-band ITPC: (*A*) left: EEG, band-pass filtered and averaged within the 8–12 Hz range at the peak electrode (i.e., CP5; blue = control participants/group, red = DD individuals/group); (*A*) right: individual phase concentration at the peak time point, frequency, and electrode (i.e., −0.35 s, 9.60 Hz, CP5), colored lines mark participants, length of lines indicates phase concentration (R); it is visible that ITPC is increased in DD individuals; (*B*) left: ITPC within the 8–12 Hz range at the peak electrode (i.e., CP5); it is visible that ITPC is increased in DD individuals; (B) right: scalp topography of the statistical difference between the DD and control groups at the peak time point (i.e., −0.35 s), frequency (i.e., 9.60 Hz); circles mark electrodes; solid circles mark electrodes where the group difference was significant at *P* < 0.05 (corrected).

For the group comparison on prestimulus alpha-band ITPC between dyslexics and controls, statistical analysis revealed a single significant cluster (cluster-sum *t*(51) = −257.71, cluster-level *P* = 0.006; peak level t(51) = −4.00, *P* = 0.006) in the time window from −0.40 to −0.15 s across the 8.80–12-Hz range with a broad scalp distribution (channels FZ, F4, F7, F8, FC3, FC4, CZ, C3, C4, T7, T8, CP5, CP6, PZ, P3, and P4; Fig. 3); alpha-band ITPC was increased for dyslexic relative to control participants.

**Table 2:** Contingency table for Cochran–Mantel–Haenszel test; participant sample was split by group, MMN median, and ITPC median

MMN	ITPC	Group	
		Control	DD
Small	Low	6	4
	High	2	14
Large	Low	14	1
	High	5	5

**Figure 4:**
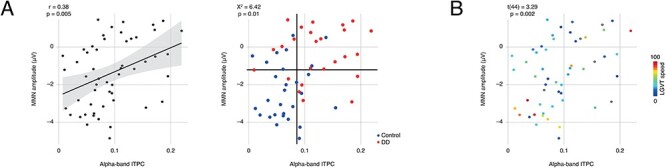
Results of post-hoc analyses: (*A*) left: Pearson linear correlation between alpha-band ITPC and amplitude of the MMN (*r* = 0.38, *P* = 0.005); right: results of individual-participant analysis: MMN and ITPC were associated across and within (DD group: 14/23 participants with small MMN and high ITPC; control group: 14/27 control with large MMN and low ITPC); solid lines mark medians of ITPC (*X*-axis) and MMN (*Y*-axis); (*B*) results from the general linear model analysis predicting reading speed from the interaction of peak ITPC (i.e., at −0.35 s, 9.60 Hz, channel CP5) and peak MMN amplitude (i.e., at 0.57 s, channel PZ); color or circle marks reading speed; it is visible that high reading speeds (i.e., red colors) associate with low ITPC and high MMN amplitudes, whereas low reading speeds associate with higher ITPC and lower MMN amplitudes (*t*(43) = 3.19, *P* = 0.003).

We had hypothesized that MMN amplitude and alpha-band ITPC might correlate, such that a high prestimulus alpha-band ITPC would associate with a small MMN ERP to the audio–visual mismatch. To follow this hypothesis, we extracted within participant the MMN amplitude at the time point and channel where the MMN ERP group difference peaked (i.e., 0.57 s, channel PZ); we also extracted ITPC values at the time point, center frequency, and channel where the alpha-band ITPC group difference peaked (i.e., at −0.35 s, 9.60 Hz, channel CP5). We then performed a Pearson linear correlation analysis, showing a significant correlation of MMN ERP amplitudes and alpha-band ITPC values across participants (*r* = 0.38, *P* = 0.005; Fig. 4*A*); k-fold cross-validation (10-folds, 10 repetitions) suggested that this correlation was reliable (*r*^2^ = 0.31, SD of root-mean-square error = 0.01).

Next, we assessed post-hoc whether individual participants with DD tend to show a concordance of a decreased MMN and an increased ITPC, while individual control participants show the opposite pattern. To this end, we created a three-way contingency table (Table 2) by splitting our sample by group (i.e., control vs. dyslexic), MMN median (i.e., above vs. below), and ITPC median (i.e., above vs. below). We then ran a Cochran-Mantel-Haenszel test (CMH; Cochran 1954). The Woolf test statistic (Woolf 1955) was nonsignificant (*Χ*^2^(1) = 6.42, *P* = 0.86), suggesting applicability of the CMH. The CMH was significant (*Χ*^2^(1) = 6.42, *P* = 0.01), stating that MMN and ITPC were associated across groups. Follow-up Fisher’s exact tests (Fisher 1962) were significant as well (control group: *P* = 1, odds ratio = 1.07; DD group: *P* = 1, odds ratio = 1.41), suggesting that MMN and ITPC were nonindependent within the group; consistent with this, 14 out of 23 participants with DD showed a small MMN in concert with a high ITPC, the opposite pattern being found in 14 out of 27 control participants (Fig. 4*A*; Table 2).

### Control Analyses

We were concerned that the group difference in prestimulus alpha-band ITPC was a mere filter-edge artifact carrying over the group difference in the subsequent MMN ERP into the prestimulus interval. To address this concern, we subtracted the ERP for the standard condition from each standard trial and the ERP for the deviant condition from each deviant trial, effectively removing the time-locked EEG, that is, the ERP (Kalcher and Pfurtscheller 1995). We then recomputed ITPC in the prestimulus interval and tested again for a group difference at the peak time point, frequency, and channel of the alpha-band ITPC effect (i.e., −0.35 s/9.60 Hz/channel CP5). The group difference stayed significant (peak-level *t*(51) = −4.00, *P* = 0.0002), suggesting that the ITPC effect was not caused by a filter-edge artifact.

**Table 3:** Results of the post-hoc correlation analysis on the behavioral data

	MMN			ITPC			MMN × ITPC		
	*t*	df	*P*	*t*	df	*P*	*t*	df	*P*
BISC	0.40	48	0.69	2.34	48	0.03	−0.67	48	0.51
DERET	−1.31	48	0.20	−1.86	48	0.07	0.38	48	0.70
BAKO	−1.20	48	0.24	−2.31	48	0.03	0.33	48	0.74
LGVT (speed)	−3.84	43	<0.001*	1.31	43	0.20	3.19	43	0.003*
LGVT (comprehension)	−2.20	43	0.03	1.07	43	0.29	1.69	43	0.10

MMN, mismatch negativity; ITPC, intertrial phase coherence; DERET, orthography assessment; BAKO, assessment of phonological awareness; BISC, preschool risk assessment of phonological awareness; LGVT, assessment of reading abilities.

^*^ *P* < 0.05, Bonferroni corrected.

When considering possible carryover effects, we became concerned that the group difference in prestimulus alpha-band ITPC could have been caused by a stationary event of the experimental procedure (e.g., the fixation cross) that might have driven an evoked response in the prestimulus interval (Klimesch et al. 2007b). To address our concern, we calculated the prestimulus ERP by averaging across standard and deviant trials; for baseline correction, we subtracted the average potential across an earlier interval from −0.75 to −0.5 s. We then tested for a group difference at the peak time point, frequency, and channel of the original alpha-band ITPC effect (i.e., −0.35 s/9.60 Hz/channel CP5). The group difference in the ERP was not significant (*t*(51) = 0.77, *P* = 0.44), suggesting that the alpha-band ITPC effect was likely not caused by a concurrent ERP.

In principle, the observed group differences could have been confounded by the group difference in trial numbers after artifact rejection. Resulting differences in temporal variance could have changed the signal-to-noise ratio of the MMN and thus its amplitude. Likewise, variance differences might have affected prestimulus phase-locking, which is calculated across trials as well. To control this, we reran the group comparisons at the MMN and ITPC peaks using one-way analyses of covariance (ANCOVAs), factoring out the individual rejection rate. Because of potential nonlinearity of rejection rates, we transformed these using the Box Cox method (Box and Cox 1964; λ = 0.20). Rejection rate predicted neither MMN amplitude (*F*(1) = 0.10, *P* = 0.75) nor prestimulus alpha-band ITPC (*F*(1) = 0.24, *P* = 0.63). There was no interaction between the group factor and artifact rejection rate (MMN: *F*(1) = 0.57, *P* = 0.45; ITPC: *F*(1) = 0.05, *P* = 0.83). The group differences stayed intact (MMN: *F*(1) = 23.90, *P* = 0.0001; ITPC: *F*(1) = 15.43, *P* = 0.0002). Together, the ANCOVA analyses suggested that the observed group differences were not related to artifact rejection rates.

### Correlation Analyses

We hypothesized post-hoc that the combination of MMN ERP and alpha-band ITPC might relate to an individual’s verbal abilities, which might be reduced in individuals with high prestimulus alpha-band ITPC and small MMN ERP amplitudes. To follow this hypothesis, we set up a general linear model in R (R Core Team 2018) separately for each standardized test. Main effects of MMN ERP amplitudes at the peak time point and channel (i.e., 0.57 s, channel PZ) and ITPC values at the peak time point, center frequency, and channel (i.e., −0.35 s, 9.60 Hz, channel CP5) were entered as predictors, as well as the interaction of the two effects. To ensure that the trending group difference in nonverbal intelligence would not confound this analysis, K-ABC scores were included into each model as a nuisance regressor. *P* values were Bonferroni-corrected for multiple comparisons. Results showed both the MMN ERP and the MMN ERP × alpha-band ITPC interaction to significantly predict reading speed according to the LGVT test (*t*(43) = −3.84, *P* < 0.001 and *t*(43) = 3.19, *P* = 0.003, respectively; Table 3 and Fig. 4*B*).

## Discussion

In a sample of school children who were tested on an audio–visual oddball experiment, we found evoked responses indicative of phonological deficits in DD to be preceded by EEG markers of deficient attention shifting. A reduced auditory MMN in individuals with DD—triggered by an incoming phoneme that mismatches a concurrent visually presented mouth gesture—is preceded and predicted by a prestimulus increase in alpha-band synchronization; jointly, the prestimulus and evoked effects predicted reading abilities. Hence, phonological deficits (i.e., reduced MMN) in individuals with DD may be secondary to deficits in the shifting of attention (i.e., increased prestimulus alpha-band synchronization) to upcoming stimuli.

Increased prestimulus alpha-band synchronization in DD individuals may result in a lack of sensitivity to incoming bottom-up information—consistent with their reduced MMN. In contrast, intact prestimulus alpha-band desynchronization in healthy participants may subserve the shifting of attention to sensitize the auditory system for the upcoming bottom-up information. Individuals with DD may thus fail to attend punctually to an upcoming auditory mismatch, possibly under distraction by a preceding stimulus. This converges on prior reports of MMN amplitude reductions when attention is distracted (Woldorff et al. 1991; Woldorff et al. 1998). MMN generators include frontal cortices (Marco-Pallarés et al. 2005; Garrido et al. 2008) that may exert auditory top-down attention via alpha-band power modulations (Thut et al. 2006, 2012; Kayser et al. 2015; Henry et al. 2017). The observation that the auditory mismatch reduces, but does not cancel out the MMN in DD individuals is still consistent with the notion that attention is not strictly necessary for MMN elicitation. The MMN occurs even during sleep (Sculthorpe et al. 2009) and in patients in coma and vegetative states (Wijnen et al. 2007; Fischer et al. 2010; for review, see Morlet and Fischer 2014). Yet, while our interpretation of the prestimulus effect in terms of temporal attention switching converges on a body of prior research, caution is at order: Our experimental paradigm did not include factors targeting attention or attention switching; neither did we include an explicit behavioral task that would target attention or attention switching; we also did not acquire standardized behavioral measure of attention or attention switching. Follow-up experiments should thus include additional factors that target attention switching more directly. In addition, deviance detection itself was not tested behaviorally, such that we should not draw overly strong conclusions about children’s behavioral discrimination abilities. Even though future MMN studies in children with DD should also consider deviance detection at the behavioral level, we are convinced that the MMN is representative for behavioral discrimination abilities (e.g., Jaramillo et al. 2000).

In principle, it could be argued that the repetitive nature of the MMN paradigm prohibits an objective interpretation of the phase-locking effect in terms of either prestimulus attention or rather continued distraction by the preceding stimulus. Yet, the prestimulus account is supported by prior proposals on the functional role of the alpha band. Here, synchronization is often proposed to index cortical inhibition, associating with decreased bottom-up information transmission and behavioral disengagement. In contrast, desynchronization indexes inhibition, associating with increased bottom-up transmission and engagement (Klimesch et al. 2007a; Jensen and Mazaheri 2010; Weisz et al. 2011). Accordingly, Maltseva et al. (2000) observed alpha-band phase reorganization to occur during the expectation of omitted visual or auditory stimuli in repetitive sequences, predicting behavioral omission detection. Hanslmayr et al. (2007) found good performance in a visual discrimination task to be predicted by decreased phase-locking (cf. Mathewson et al. 2009; Mathewson et al. 2011). Weisz et al. (2014) found reduced prestimulus power to predict conscious perception of near-threshold somatosensory stimuli. Direct correlations between prestimulus activity and evoked potentials are attested both for early visual components (<200 ms; Iemi et al. 2019) and later components related to cognitive processing in vision and somatosensation (>175s and >140 ms, respectively; Becker et al. 2008; Reinacher et al. 2009; for discussion, see Mathewson et al. 2011)—consistent with the timing of the current MMN response (for review, see Näätänen et al. 2019).

The current study suggests alpha-band phase-locking as a possible electrophysiological substrate of deficient attention shifting in individuals with DD (Hari and Renvall 2001; Krause 2015), suggesting that their phonological processing deficits (Snowling 1998; Goswami 2015; Peterson and Pennington 2015) may be secondary. Sluggish attention shifting to auditory information arriving at a quick pace may result from a prolonged auditory processing time window, resulting in the sampling of prolonged input chunks (Hari 1995; Wright et al. 1997; Hari and Renvall 2001; for review, see Krause 2015). Under this view, the succession of auditory stimuli in our MMN paradigm may have outrun the auditory sampling frequency in individuals with DD. Alternatively, our audio–visual MMN paradigm may rather have overburdened audio–visual attention shifting in individuals with DD. In the current paradigm, video sequences of mouth movements still continued after the offset of the auditory stimulus, providing an even-shorter time window to disengage from visual information and engage with the upcoming auditory information. It has been discussed that deficient auditory attention shifting is really a consequence of an visual–auditory attention shifting deficit. Behavioral work and functional imaging studies observed deficient attention shifting in DD not only in the auditory domain (Raschle et al. 2013), but also in the visual, spatial, and visuo–spatial domains; in particular, switching from the visual to the auditory domain during the transition from written text to speech sounds associates with DD (Franceschini et al. 2012, 2013; Harrar et al. 2014; Ruffino et al. 2014; for review, see Krause 2015; Gori et al. 2016; Franceschini et al. 2017; Franceschini and Bertoni 2019). Further oddball experiments could help to dissociate the auditory and amodal accounts.

The use of ITPC in the current study differs from some prior work that has proposed induced alpha-band power as a measure of prestimulus excitability. In the literature, low alpha-band power is thought to measure increased phase synchronization (e.g., Hanslmayr et al. 2007), because alpha-band activity is mostly caused by inhibitory interneurons (Weisz et al. 2011). Low power, resulting from a high phase synchronization, thus associates with high neuronal excitability (Lakatos et al. 2016), which is beneficial for processing (Wöstmann et al. 2019). The use of ITPC is compatible with this idea: When phase varies across trials, excitability is more uniform over time; this should increase temporal flexibility in reacting to upcoming stimuli and thus enhance subsequent ERPs. In contrast, phase consistency should reduce flexibility, increasing temporal variability of evoked potentials across trials, decreasing the ERP (Klimesch et al. 2007b).

In sum, we here provide evidence that aberrant prestimulus alpha-band synchronization is a substrate of an underlying attention shifting deficit in individuals with DD. Prestimulus oscillations may be a previously overlooked biomarker of attention shifting deficits in DD. Because the current study is restricted by the use of an audio–visual paradigm and the focus on a sample of school children, we foresee great potential for the joint assessment of prestimulus oscillations and evoked responses in infant and adult age groups, as well as for longitudinal and translational studies.

## Notes

*Conflict of Interest*: None declared.
